# Editorial: Advances in non-invasive brain stimulation techniques

**DOI:** 10.3389/fnins.2024.1524097

**Published:** 2024-12-20

**Authors:** Mark H. Myers, Gahangir Hossain

**Affiliations:** ^1^Department of Anatomy and Neurobiology, University of Tennessee Health Sciences Center, Memphis, TN, United States; ^2^Department of Information Science, University of North Texas, Denton, TX, United States

**Keywords:** transcranial magnetic stimulation, transcranial direct current stimulation (tDCS), transcranial magneto-acoustic stimulation (TMAS), transcranial alternating current stimulation (tACS), photobiomodulation (PBM)

Non-invasive brain stimulation offers a painless and safe approach to neurological rehabilitation, providing minimal side effects, and has been used by thousands of people worldwide. Non-invasive brain stimulation modulates the brain's excitability to aid in treating neurological disorders. The latest work has moved well beyond implementations of transcranial magnetic stimulation/transcranial direct current stimulation (TMS/tDCS) and deep brain stimulation (DBS) techniques, such as repetitive transcranial magnetic stimulation (rTMS), transcranial magneto-acoustic stimulation (TMAS), transcranial alternating current stimulation (tACS), transcutaneous auricular vagus nerve stimulation (taVNS), and peripheral electrical stimulation (PES). Previous intervention techniques, which were applied to alcohol addiction, have moved on to stroke intervention, Alzheimer's, epilepsy, depression, migraine, and tremor alleviation, such as in Parkinson's disease. Exciting research in eSports for performance improvement and remote non-invasive brain stimulation is now being considered. The Frontiers in Neuroscience Research Topic, “*Advances in non-invasive brain stimulation techniques*”, has 19 articles covering the latest research in this exciting area.

Transcranial magnetic stimulation has been used in the treatment of Parkinson's disease (PD). In an analysis of 1,894 papers between 1991 and 2022, PD was used as a topic and analyzed. The ranking of publications was found in the following order: United States (293), Italy (184), China (151), England (119), and Canada (100). An analysis of institutions found that the most publications were from the University of Toronto (95), Harvard University (74), and the Sapienza University of Rome (63) (Wei et al.).

A magnetic pulsing approach has been applied to aid those with difficulty swallowing (dysphagia). A therapy involving motor-evoked potentials that applies a 5 ms session-monophasic quadripulse magnetic stimulation (QPS5) was studied. A stimulation that applies 5 ms session sets of four burst trains has been found to facilitate bilateral mylohyoid MEPs enormously. These studies provide insight into motor-evoked potential therapy and the central nervous system response to dysphagia (Fujiki et al.).

TMS has been found to modulate the excitability and plasticity of ascending corticospinal respiratory pathways. Both rTMS and tDCS can regulate respiratory functionality networks in healthy and diseased brains. Phrenic motor neurons (PMN) and the supplementary motor area (SMA) may also be critical regulatory areas. Diaphragmatic motor-evoked potentials (DiMEPs) decreased on the non-injured side but not the injured side after an injury, indicating increased excitability of PMNs on the ipsilateral side. In the manuscript, the authors review two cases where one patient is administered peripheral electrical stimulation (PES) on the pectoral and abdominal muscles and anode tDCS on the SMA. The application of sensory PES stimulation is inhibitory in this instance. For the second patient, they received excitatory PES on the abdominal muscles. The application of PES on the abdominal muscles enables an excitatory motor response. Both patients had spinal cord injuries with long-term tracheotomy. The results from this study demonstrate that the SMA, under both TMS and tDCS, may be considered for respiratory regulation (Lv et al.).

Repetitive transcranial magnetic stimulation (rTMS) is a practical post-stroke cognitive impairment (PSCI) intervention. After a stroke, increased neural excitability of the unaffected hemisphere and heightened inhibition of the damaged hemisphere are likely to occur. Conversely, reduced inhibition appears in the damaged hemisphere of the skull. Cortical homeostasis is restored by applying high frequency (HF)-rTMS to the affected side to reduce cortical excitability and then using low frequency (LF)-rTMS on the healthy side of the bilateral dorsolateral prefrontal cortex DLPEF (Wang Y. et al.). Another study featuring stroke rehabilitation involves intermittent theta-burst stimulation on the upper limbs for motor recovery. Performance improvement has been found for stroke patients involving improved motor recovery from impairment and better quality of life to perform daily activities (Chen et al.).

Repetitive high-frequency transcranial magnetic stimulation applied over consecutive 2- and 25-s intervals increased cortical excitability. Increased alpha activity was seen in the central regions of the brain. Enhanced functionality was also exhibited in the same central areas toward other brain areas (Jin et al.).

Transcranial magneto-acoustic stimulation (TMAS) has been found to improve the spatial memory deficits of the APP/PS1 mouse model of Alzheimer's disease (AD). The technique involves ultrasound stimulation within a static magnetic field. TMAS was applied to mice for several weeks and then evaluated using *in-vivo* electrophysiology and the Morris water maze (MWM) task. Through analyzing brain oscillations, TMAS improved the performance of MWM-related spatial cognitive functions in the mouse model of Alzheimer's disease (Zhang S. et al.).

Transcranial-focused ultrasound (FUS) offers higher spatial resolution when positioning the radius to targeted brain tumors. This method also provides improvement over targeted depth therapies. Through this therapy, focused ultrasound treatment can be delivered with higher precision (Gao et al.).

Several approaches have been considered for the treatment of fibromyalgia. Transcranial alternating current stimulation (tACS) applies a sinusoidal alternating current to modulate an electric field within the brain. This approach parallels the induced changes of cortical activity in the brain and modulates long-term synaptic plasticity. Additionally, transcranial-focused ultrasound (FUS) provides both spatial precision and deep penetration of brain regions involving ultrasound pulses that have been proven effective in the treatment of fibromyalgia (Zhang J.-H. et al.).

Vagus nerve stimulation (VNS) has traditionally been utilized for the treatment of intractable instances of epilepsy. Transcutaneous auricular VNS (taVNS) has also been applied to patients who have stimulation delivered to the auricular branch of the vagus nerve (ABVN). taVNS could be used for epilepsy, depression, and migraine. taVNS has even been found to elevate cerebral blood flow through repetitive auditory stimuli (RtAS) in patients with consciousness disorders (Wang L. et al.).

Transcranial alternating current stimulation (tACS) is an emerging tool for improving cognitive response and reaction times in Electronic Sports (eSports) and Shooting Games. By modulating oscillatory cortical networks, tACS can alter regional and more extensive network connectivity, decreasing reaction time in visual spatial attention and improving quick classification tasks, such as exact aiming. High-definition HD-tACS has exhibited an improvement in cognitive responses through the reduction of distraction during task performance. Additionally, high coherence between frontal and parietal lobes was also demonstrated. This approach may be carried onto rehabilitation training in patients with cognitive deficits (Jiao et al.).

Transcranial alternating current stimulation may be considered for patients with cerebellar damage, affecting areas such as timing, attention, memory, and language, and those who suffer from tremor-type conditions. Transcranial alternating current stimulation can induce Purkinje cell spike synchrony, which may be helpful for patients with cerebellar damage. This approach may address deficits in language, memory, and attention (Kang et al.).

Other areas of experimental stimulation include cortico-cortical paired associative stimulation (ccPAS), low-intensity pulsed ultrasound (LIPUS), transcranial Photobiomodulation (tPBM), Transcranial Burst Electrical Stimulation (tBES), and Rapid X-ray based genetically targeted (X-genetic) manipulation. This work highlights remote intervention, low power systems to reduce patient risk, generalized pain reduction, merged interventions such as tBES, and genetic modulation. This work is still in its infancy but shows the next advancement in neurological intervention and cognition research.

Cortico-cortical paired associative stimulation (ccPAS) differs from paired associative stimulation (PAS), which uses a dual-coil TMS approach to apply repetitive paired-pulse stimulations over two cortical regions. ccPAS is believed to induce spike-timing dependent timing over a cortico-cortical connection, such as primary motor cortex to primary motor cortex (M1), ventral premotor cortex to primary motor cortex, supplementary motor area to primary motor cortex, and posterior parietal cortex (PPC)-M1. ccPAS over the ventral premotor cortex (PMv) and M1 tapped into motor tasks could increase the motor-evoked potential in healthy adults. ccPAS over the PMv and M1 could decrease the motor-evoked potential in healthy adults. These effects may be due to PMv-M1 glutamatergic projections, which activate the local inhibitory circuits more than the excitatory circuits within the M1. ccPAS may be a therapeutic solution for a host of various neurological issues such as stroke, Parkinson's disease, and major depressive disorders that involve brain connectivity and networks (Zhang).

Low-intensity pulsed ultrasound (LIPUS) has been used to promote nerve regeneration and repair. Rapamycin (mTOR) signaling pathways are known as neurological regulators. Applying LIPUS treatments has led to increased neurite length and mammalian target of rapamycin (mTOR) signaling pathways, where aberrant mTOR signaling equates to abnormal neuronal function. Additionally, LIPUS is considered an application to stimulate neuroblastoma cells to enable neurite outgrowth for neurological diseases such as Alzheimer's, Parkinson's, and many other areas (Ye et al.).

Transcranial Photobiomodulation (tPBM) is a method administered to the cerebral cortex that has been shown to enhance human cognition and attentional performance and treat Alzheimer's and Parkinson's disease. The treatment involves the application of a 1,064-nm continuous-wave laser onto the frontal region of the brain (Fp2) without any contact. The left parietal region has increased alpha activity during a tPBM session. This application may be a non-evasive method that can be applied to many neurological applications (Truong et al.).

Deep brain stimulation is a technique that involves implanting electrodes into subcortical areas of the brain to administer electrical currents. It is considered minimally invasive, considering other surgical methods. A study focusing on deep brain stimulation (DBS) for the treatment of chronic pain considers two significant areas: chronic pain treatment (DBS-P) and DBS for other indications (DBS-O), such as Parkinson's disease or dystonia. The analysis included 966 patients with chronic pain who underwent DBS (340 for DBS-P and 625 for DBS-O). An average pain reduction of ~48% for the DBS-P group and ~60% for both groups was found. Additional research is needed to optimize the outcomes of DBS's utility for chronic pain (Shaheen et al.).

A closed-loop neurostimulation simulator has been developed based on stimulation sets through observations. Through this simulator, researchers may find that tDCS therapy can be optimized for more extended periods at higher yields without yielding any adverse health risks. This work presents a brain stimulation simulator to provide several techniques applied to a range of clinical issues, such as Parkinson's disease (PD) or obsessive-compulsive disorders (Wahl et al.).

Transcranial Burst Electrical Stimulation (tBES) combines direct current (DC) and theta burst stimulation (TBS) for brain neuromodulation. In a study by Nguyen et al., tBES (–) stimulation caused an elevation in GAD-65 expression, which has been used as a marker for excitatory and inhibitory neuronal activity in rodent brains. Compared to the baseline and sham state, a decrease in MEPS from tBES (–) stimulation was found. tBES has shown promising results in modulating motor cortical excitability and treating neurological disorders (Nguyen et al.).

Rapid X-ray-based genetically targeted (X-genetic) manipulation of cellular electrical activity for intact-behaving animals has been demonstrated in *Caenorhabditis elegans*. Transgenic expression of LITE-1 in *C. elegans* muscle cells resulted in paralysis and egg ejection responses to stimulation. This experiment shows that LITE-1 could be an X-ray-sensitive receptor in *C. elegans*. This study identifies an X-ray receptor protein that can be trans-genetically expressed in cell types to acutely control the activity of those cells using X-rays and demonstrates X-genetic control of cellular electrical activity. These findings suggest a minimally invasive approach to neuromodulation using transcranial X-ray signals for the manipulation of neural activity in mammals (Cannon et al.).

Altogether, neurological pathologies and the approach to non-invasive intervention is a multi-pronged, multi-layered approach. Electrical intervention (direct/alternating), magnetic, acoustic, ultrasound, paired-pulsed, electrical bursts, photobiomodulation, rapid X-ray-based, and deep brain stimulation have their merits in solving various neurological diseases. Parkinson's and stroke intervention present the initial beachhead to provide early results through this intervention and prove its efficacy. There are specialized techniques that focus on a specific condition, such as respiratory rehabilitation for spinal injury patients, directed treatments for dysphagia, and further bolstering the cognitive capabilities of those who have Alzheimer's disease. Non-invasive techniques are now considered an approach to higher spatial precision and regulation depth to brain tumors. What was considered improbable regarding rapid memory and learning enhancement is now in practice for improving cognitive functions, such as in Electronic Sports. High-definition HD-tACS has been shown to reduce visual spatial attention distraction and enhance task performance during stimulation for eSports participants.

Furthermore, it is now being considered as a rehabilitation approach. Even remote intervention is possible while maintaining low power incidence to the patient. Various research articles highlight generalized pain reduction, merged interventions, and genetic modulation. This work represents research that may find itself very quickly in clinical environments. This field of neurological intervention and rehabilitation is advancing in many fascinating directions. The most interesting non-invasive measures are just around the corner!

